# Inflammatory bowel disease: an overview of Chinese herbal medicine formula-based treatment

**DOI:** 10.1186/s13020-022-00633-4

**Published:** 2022-06-18

**Authors:** Shuo Yuan, Qi Wang, Jiao Li, Jia-Chen Xue, You Li, Huan Meng, Xiao-Ting Hou, Ji-Xing Nan, Qing-Gao Zhang

**Affiliations:** 1 Key Laboratory of Natural Medicines of the Changbai Mountain, Ministry of Education, College of Pharmacy, Yanbian University, Yanji, 133002 Jilin China; 2 Present Address: Chronic Disease Research Center, Medical College, Dalian University, Dalian, 116622 Liaoning China; 3 Department of Immunology and Pathogenic Biology, Yanbian University College of Basic Medicine, Yanji, 133002 Jilin China

**Keywords:** Autophagy, Chinese herbal medicine formula, Immunity, Inflammatory bowel disease, Intestinal mucosal barrier, Oxidative stress

## Abstract

Inflammatory bowel disease (IBD) is a chronic recurrent inflammatory disease of the intestine, including Crohn’s disease (CD) and ulcerative colitis (UC), whose etiology and pathogenesis have not been fully understood. Due to its prolonged course and chronic recurrence, IBD imposes a heavy economic burden and psychological stress on patients. Traditional Chinese Herbal Medicine has unique advantages in IBD treatment because of its symptomatic treatment. However, the advantages of the Chinese Herbal Medicine Formula (CHMF) have rarely been discussed. In recent years, many scholars have conducted fundamental studies on CHMF to delay IBD from different perspectives and found that CHMF may help maintain intestinal integrity, reduce inflammation, and decrease oxidative stress, thus playing a positive role in the treatment of IBD. Therefore, this review focuses on the mechanisms associated with CHMF in IBD treatment. CHMF has apparent advantages. In addition to the exact composition and controlled quality of modern drugs, it also has multi-component and multi-target synergistic effects. CHMF has good prospects in the treatment of IBD, but its multi-agent composition and wide range of targets exacerbate the difficulty of studying its treatment of IBD. Future research on CHMF-related mechanisms is needed to achieve better efficacy.

## Background

Inflammatory bowel disease (IBD) is non-specific colitis, mainly including ulcerative colitis (UC) and Crohn’s disease (CD), with symptoms such as abdominal pain, diarrhea, and weight loss [1]. Currently, the prevalence of IBD is on the rise worldwide, with a prevalence of 10–20% in developed countries and more severe in developing countries [2, 3]. Pharmacological treatment of IBD in modern society mainly uses drugs such as 5-aminosalicylates and immunosuppressive agents. However, these drugs have side effects such as drug resistance and intestinal flora dysbiosis, which seriously affect the prognosis of patients’ lives [4]. Therefore, it is vital to investigate the pathological mechanism of IBD and find new targets and drugs for IBD treatment. The interaction between genetic and environmental factors, immune dysfunction, intestinal flora dysbiosis, and impaired intestinal mucosal barrier function is currently the leading causes of IBD development.

With the continuous development of the global pharmaceutical industry, Traditional Chinese Medicine has received more and more attention. Most Chinese Herbal Medicine has a long history of clinical experience in the treatment guided by Chinese medical theory. Chinese Herbal Medicine's development and exploitation procedure are the opposite of chemical drug research, which mainly starts with clinical practice screening to identify candidate compounds and then clinical validation to enter the new drug development track for clinical research, production, and marketing [5]. The Chinese Herbal Medicine Formula (CHMF) has certain specificities and advantages compared with some Western medicines. CHMF is a formula composed of two or more medicinal flavors, with relatively prescriptive processing and usage methods, for relatively definite disease evidence and is the main component of Traditional Chinese Medicine prescriptions. CHMF has a complex chemical composition and pharmacological effects with multi-target medical characteristics [6].

It has been shown that the co-administration of *Indirubin* and *Isatin*, the co-administration of *Astragalus membranaceus* polysaccharides, and *Codonopsis pilosula* polysaccharides, as well as the administration of Fuzi-Ganjiang, can improve clinical symptoms in UC mice [7–9]. The remarkable therapeutic advantages of Chinese medicine for IBD have received increasing attention from scholars, and this article describes the progress of basic research achieved by CHMF in the treatment of IBD in recent years. The current use of CHMF for IBD treatment is mainly focused on improving intestinal mucosal barrier function, immunomodulation, and regulating oxidative stress.

## Intestinal mucosal barrier

Patients with IBD have a damaged intestinal mucosal barrier, leading to abnormal cytokine secretion, intestinal mucosal atrophy, permeability changes, and intestinal flora displacement [4]. This may lead to recurrent inflammation and aggravation of inflammation. The intestinal mucosal barrier comprises four parts: mechanical barrier, chemical barrier, immune barrier, and biological barrier. If one of the barriers is damaged, it will lead to the destruction of the intestinal mucosal barrier, resulting in impairment of its function, induction of intestinal infection, and the occurrence of diseases such as IBD.

### Intestinal mucosal mechanical barrier

The mechanical barrier is the intestinal mucosal epithelial structure, including the intestinal epithelial cells (IECs) and the connections between IECs, which absorb nutrients and exclude harmful substances outside the barrier. The intestinal mucosal epithelial cells and the tight junction (TJ) between the cells form the intestinal mucosal mechanical barrier. The integrity of the intestinal mucosal mechanical barrier is determined by the composition and function of IECs and TJs [10]. The disruption of TJ infiltrates harmful luminal molecules and disrupts the intestinal mucosal immune system and inflammation. Therefore, TJ can act as a trigger for the development of intestinal and systemic diseases [11]. The TJ comprises a family of cytoplasmic proteins, like ZO (zonula occludens proteins), transmembrane proteins (tricellulin, nectin, junctional adhesion molecules, occludin, and claudins), and cytoskeletal structures together. Occludin and claudins can regulate the function of the intestinal mucosal mechanical barrier by affecting TJ permeability [12]. Targeting the restoration of TJs integrity may become an effective way to treat and prevent IBD.

In dextran sulfate sodium (DSS)-induced UC mice, the QingBai decoction, Huang-Lian-Jie-Du Decoction, and Huangqin Decoction increased the expression of ZO-1 and Occludin in colonic mucosal [13–15]. Fang investigated the effect of Qing Hua Chang Yin on the loss of intestinal epithelial barrier integrity induced by lipopolysaccharide (LPS) in vitro using a Caco-2 cell model and found that Qing Hua Chang Yin could upregulate the mRNA and protein expression levels of Claudin-1 [16]. In addition, Sijunzi Decoction can also upregulate the level of Claudin-2 in the colon tissue of rats induced by a 2,4,6-trinitrobenzene sulfonic acid (TNBS) in vivo as an intestinal barrier protector [17].

### Intestinal mucosal chemical barrier

The mucosal chemical barrier is less reported, but it also plays an essential role in IBD. The loose mucus layer covering the intestinal epithelial cells and the mucin in the mucus together constitute the intestinal mucosal chemical barrier [18]. Mucins can prevent pathogenic bacteria from invading and adhering to the intestine. Huang-Lian-Jie-Du Decoction can protect the intestinal mucosa by increasing the secretion of mucins [14].

### Intestinal mucosal microbial barrier

In recent years, intestinal flora disorders have become an essential factor in the pathogenesis of IBD. The micro spatial structure of the intestinal commensal bacteria and the host constitutes the intestinal mucosal microbial barrier. Intestinal flora can interact with cells to promote barrier function through nutrient acquisition, energy and metabolic regulation, and cell proliferation [19]. The relationship between the intestinal flora and the organism's autoimmune system coexists and influences each other. The imbalance of intestinal microecology can lead to various inflammatory and metabolic diseases in the body [20]. It has been shown that during the active phase of IBD, there is intestinal flora dysbiosis and decreased species diversity within the organism [21]. CHMF can regulate intestinal flora disorders and maintain the homeostatic balance of intestinal flora [22]. Huang-Lian-Jie-du Decoction, Huangqin Decoction, and *Pyungwi-san* can restore the balance of intestinal flora in UC mice by inhibiting the growth of intestinal pathogens and preventing the reduction of beneficial bacteria [23–25]. In DSS-induced UC rats, the ratio of Bacteroidetes to Firmicutes was elevated. This ratio decreased to normal levels with the use of Huai Hua San [26]. It has also been suggested that IBD is associated with Clostridium difficile (CDD) infection, resulting from dysbiosis of the intestinal flora. In contrast, *Pyungwi-san* normalizes the abundance ratio of Firmicutes/Bacteroidetes in the intestine and has some protective effect against DSS + CDD-induced colitis, which may be achieved by restoring the balance of the intestinal microbial community [27].

IBD can cause abnormal metabolic regulation in vivo. The resident intestinal flora can suppress the expression of pro-inflammatory cytokine genes by secreting short-chain fatty acids (SCFAs), vitamins, and other beneficial active metabolites [28]. Several metabolic pathways, including amino acids, fatty acids, and bile acids, are perturbed in IBD patients. Metabolomics characterizes the overall and dynamic changes in the type and concentration of endogenous small-molecule metabolites (e.g., amino acids, lipids, nucleosides) in the organism when disturbed by disease or drugs [29]. Subsequently, metabolomics can be correlated with genomic and proteomic results to facilitate the systematic study and comprehensive interpretation of disease mechanisms or drug mechanisms of action [30, 31]. Therefore, metabolomics is used to characterize the endogenous metabolic profiles of IBD patients and to reveal the critical metabolic pathways that are perturbed during the development and progression of IBD. It has important implications for the in-depth investigation of the pathogenesis of IBD and the search for new therapeutic targets for IBD. Hong analyzed the metabolic profile of UC rats by LC–MS/MS and identified a total of 36 differential metabolites involved in multiple metabolic pathways. Compound Sophorae Decoction could affect multiple metabolic pathways in TNBS-induced UC rats, thus diminishing their pathophysiological symptoms and obtaining positive therapeutic effects [32]. Huankuile Suspension inhibits inflammatory response and regulates bile metabolism, pyrimidine metabolism, purine metabolism, glutathione metabolism, and citric acid cycle in UC rats [33]. Huang-Lian-Jie-du decoction alleviates UC in mice by regulating arachidonic acid metabolism and glycerophospholipid metabolism [34]. *Astragalus membranaceus* polysaccharides and *Codonopsis pilosula* polysaccharides could upregulate the effects of isovaleric and butyric acids in SCFAs to improve clinical symptoms in mice with colitis [9]. Huangqin Decoction plays a crucial role in normalizing metabolic disorders by regulating the levels of amino acid, lipid, and fatty acid markers in UC rats [35, 36]. Rhubarb Peony Decoction increased the number of butyric acid-producing *Butyricicoccus pullicaecorum* and the level of SCFAs to restore intestinal function in UC mice. The dynamic changes of intestinal flora and host co-metabolites were detected by metabolomics to clearly demonstrate the metabolic status of intestinal flora in the host, which may provide clues and directions for studies such as the drug treatment mechanism IBD [37].

### Intestinal mucosal immune barrier

Gut-associated lymphoid tissue (GALT), secretory antibodies, and mesenteric lymph nodes (ETC) constitute the intestinal mucosal immune barrier, which can respond to antitoxins, antigens, and potentially harmful organisms [38]. GALT produces IgA, which forms the antigen complexes with antigenic material, binds to receptors on M cells, and antigens are transferred to the lamina propria and then presented to dendritic cells (DCs). Inflammatory DCs play an essential role in the pathogenesis of IBD, and Sishen Pill can modulate the interaction between inflammatory DCs and the gut microbiota to treat DSS-induced colitis [39].

## Immunity

### T lymphocytes

The exact mechanism of cellular dysfunction leading to IBD is not fully understood. However, modulating the function of immune cells could be a powerful tool in the treatment of inflammatory diseases. The adaptation of T lymphocytes to the intestinal environment requires constant differentiation between natural stimuli from commensal flora, natural stimuli from food, and pathogens that need to be removed. T lymphocytes in the gut can be activated by environmental and other factors, causing genetic variants of intestinal defense defects or tolerance disruptions, such as intestinal infections or flora imbalances, triggering multiple immune disorders [40]. Therefore, T lymphocytes are the critical site of drug action for IBD treatment. The intestinal inflammatory infiltrate is mainly composed of CD4^+^ T cells, regulatory T cells (Tregs), and main memory T Cells (Tcm) [41]. There are several specific subpopulations of T helper cells: Th1, Th2, Th9, Th17, Th22, T follicular helper (Tfh), and several auto Tregs [41]. Th17 cells play an essential barrier role in the skin and intestinal mucosa. The resistance to bacteria and fungi is an essential driver of autoimmune disease, which often exacerbates disease when triggered in the autoimmune environment. The differentiation of Th17 is mainly driven by interleukin (IL)-6 and transforming growth factor (TGF)-β, which is further stabilized by paracrine signals such as IL-23 and IL-1β [42]. It is now generally accepted that the solid anti-inflammatory CD4^+^ T-cell, Tregs, are abundant in the intestine [43]. In DSS-induced UC mice, Huangqin Decoction was able to increase the number of Tregs to alleviate the inflammatory effects [15]. The role of Tregs cells is mainly to suppress inflammation by suppressing T cells and regulating other immune cells in their environment, especially interconnected with Th17 cells in differentiation, which together maintain the body's balance of the immune microenvironment.

Once this balance is disrupted, multiple autoimmune diseases, including IBD, can occur [44]. In exploring the relationship between T lymphocytes and IBD, it has been found that Gegen Qinlian Decoction could restore the balance of Treg and Th17 cells in the colonic tissue of UC mice [45]. In TNBS-induced mouse mesenteric lymph node lymphocytes and lamina propria monocytes, Qingre Zaoshi Liangxue Decoction decreased the proportion of Th17 cells and increased the proportion of Treg cells [46]. Compound Sophorae Decoction can regulate the percentage of Th17 and Treg cells in the mesenteric lymph nodes of UC mice [47]. Furthermore, Bawei Xileisan restored the balance of Th17/Treg in monocytes [48]. The role of Tfh cells is to participate in B-cell differentiation and play a role in immunoglobulin production and the formation of lymphocyte tissue-growing centers. Sishen Pill effectively treated chronic colitis by regulating Tfh cell differentiation and function to treat IBD [49].

### NF-κB

The nuclear factor kappa B (NF-κB) family includes NF-κB1 (p50 and precursor p105), NF-κB2 (p52 and precursor p100), and related factor A, nuclear factor c-Rel, and related factor B. They all have a Rel homologous structural domain at the N terminus, which dimerizes with the DNA sequence that specifically binds to DNA sequences and activates or represses downstream transcription [50]. In the absence of external signal stimulation, the above proteins form homodimers or heterodimers and are strictly inhibited by the inhibitor of the NF-κB (IκB) family. They are regulated by the inhibitor of the NF-κB kinase (IKK) complex [51]. NF-κB is a crucial regulator of inflammation and can be activated by various stimulatory factors. The degradation of IκB translocates NF-κB to the nucleus and mediates the transcription of various target genes. Several pro-inflammatory factors encoded by the NF-κB signaling pathway promote inflammation-associated tissue damage and are associated with tumor development [52]. Huangkui Lianchang Decoction inhibits the NF-κB signaling pathway in DSS-induced ulcerative colitis [53]. Jian-Pi Qing-Chang Decoction and Tou Nong San can improve mucosal inflammatory response and intestinal epithelial barrier function through the NF-κB pathway. Also, their anti-inflammatory effects are associated with NF-κB regulation [54–57].

TOLL-like receptors (TLRs) are cellular transmembrane receptors in the natural immune system. TLRs can bind to pathogen recognition pattern molecules, activate downstream signaling molecules, and ultimately trigger the expression of inflammatory mediators, serving as a link between natural immunity and acquired immunity. TLR4 is a subtype of the TOLL receptor family, and myeloid differentiation factor 88 (MyD88) is the main junction protein in the TLR4 signaling pathway. The interaction between TLR4 and MyD88 ultimately mediates the innate immune response and inflammatory response against pathogenic bacteria [58]. It was found that the TLR4 mediated signaling pathway is involved in the development of UC. TLR4 is a transmembrane receptor in the natural immune system, which recognizes the corresponding ligand and then binds to it to initiate signal transduction, leading to NF-κB activation and release of intestinal inflammatory mediators [59]. Gegen Qinlian Decoction can inhibit the TLR4/NF-κB signaling [60]. Kuijieyuan Decoction ameliorates intestinal barrier damage in ulcerative colitis by affecting TLR4-dependent NF-κB signaling [61].

Myosin light chain kinase (MLCK) is a calmodulin-dependent serine/threonine-specific protein kinase whose primary function is to phosphorylate myosin light chain (MLC) and activate myosin heavy chain adenosine triphosphatase. MLCK mediates the sliding of skeletal protein microfilaments, causing cell contraction and eventually the formation of cellular gaps [62]. A variety of signaling pathways are involved in the activation of MLCK, mainly through the mitogen-activated protein kinase (MAPK) pathway of extracellular regulated protein kinase (ERK) 1/2, P38, c-Jun N-terminal kinase (JNK) pathway, as well as inflammation-related pathways involved in cell proliferation and apoptosis [63]. In TNBS-induced UC rats, Bu-Zhong-Yi-Qi Granule can regulate the secretion of some inflammatory cytokines and improve TJ integrity through TLR4/NF-κB/MLCK pathway [64]. Sijunzi Decoction could reduce the levels of NF-κB p65 and MLCK [17]. In addition, Shen-Ling-Bai-Zhu-San attenuated DSS-induced UC mice via the MAPK/NF-κB signaling pathway [65]. NEMO is encoded by the B cell-encoded κ light chain polypeptide repressor gene (IκBKG) [66], and in 1998, Brott cloned the mammalian homolog of the Nemo gene named Nemo-like kinase (NLK) [67]. It has been confirmed that NLK is a highly conserved MAPK-like kinase during evolution and has a significant reference value for disease prognosis [68]. Sishen Pill can inhibit NF-κB activation by suppressing the NEMO/NLK signaling pathway and plays a vital role in treating chronic colitis [69].

The assembly of NOD-like receptor protein 3 (NLRP3) requires the involvement of the sensor NLRP3 pattern recognition receptor, apoptosis-associated speck-like protein containing a CARD (ASC), and the effector protein caspase-1 are involved, which widely presented in various immune cells when the disease occurs [70]. Microbial molecules require the activation of NLRP3 or some signaling through NF-κB proteins, which induce NLRP3 to indirectly promote the expression of the assembled inflammasome complex [71]. Both Jiaweishaoyao Decoction and *Pyungwi-san* can inhibit the NLRP3 inflammasome, and the NF-κB pathway to alleviate DSS-induced UC [72, 73].

PI3K is an intracellular phosphatidylinositol kinase. Upon the activation of PI3K, PIP3 acts as a second messenger and binds to regional proteins of Akt. This leads to the acquisition of Akt activity, which is involved in cell growth, cell development, and apoptosis through the regulation of downstream proteins [74]. Akt is a serine/threonine kinase, also known as protein kinase B or PKB, which is a target of action downstream of the PI3K pathway, and its anti-apoptosis mechanism is phosphorylation of target proteins through multiple downstream pathways. The activated Akt is involved in activating and inhibiting multiple targets after phosphorylation, enabling cell survival, growth, and proliferation through multiple mechanisms [75]. Zuojin Pill can modulate the crosstalk between intestinal microbes and Treg cells to attenuate DSS-induced colitis through PI3K/Akt signaling pathway [76]. Sishen Pill alleviates DSS-induced colitis, which may be related to inhibiting the PI3K/Akt signaling pathway [77]. Xianglian Pill can block the activation of the PI3K/Akt/mTOR pathway, inhibit the secretion of pro-inflammatory cytokines, and repair the dysfunction of the intestinal epithelial barrier to enhance autophagy [78]. Upon activating the PI3K/Akt pathway, activated Akt inhibits phosphorylation degradation of protein IκB kinase by enhancing NF-κB, which subsequently leads to NF-κB activation. Huangqin Decoction can improve DSS-induced colitis by modulating the intestinal microbiota and inhibiting the PI3K/Akt/HIF-1α and NF-κB pathways [79]. Kuijieyuan Decoction ameliorates intestinal barrier damage in ulcerative colitis by affecting TLR4-dependent PI3K/AKT/NF-κB signaling pathway [61].

### JAK2/STAT3

The JAK/STAT3 signaling pathway is composed of Janus kinase (JAK), tyrosine kinase receptor, and Signal Transducer and Activator of Transcription 3 (STAT3), which are essential in cell growth, cell proliferation, cell invasion, cell metastasis, and regulation of apoptotic processes [80]. The STAT family, a group of intracellular proteins that signal and activate transcriptional functions, contains seven members (STAT1-4, 5A, 5B, and 6). The JAK2/STAT3 signaling pathway is an important pathway mediating the signaling of numerous cytokines and inflammatory mediators, closely associated with the expression of inflammatory immune factors associated with tumor necrosis factor-α (TNF-α), IL-6, IL-17, and IL-22 [81]. The activation of JAK2 will phosphorylate tyrosine residues, which are later bound to the receptor and phosphorylated by STAT3. Phosphorylated JAK2 forms a dimer or heterodimer with phosphorylated STAT3 and ectopic to the nucleus, affecting the transcription of downstream genes and the expression of inflammatory factors [82]. Gegen Qinlian Decoction and Huanglian Jiedu Decoction reduce inflammation by inhibiting JAK2/STAT3, decrease inflammation by inhibiting JAK2/STAT3 signaling, and has a protective effect on UC [45, 83]. Qingre Zaoshi Liangxue Decoction, Pien Tze Huang, and Baitouweng Decoction significantly improved the inflammatory symptoms in mice with acute colitis, and the latent mechanism may be related to various signaling pathways, including regulation of gut microbiota and inflammatory signaling pathways, such as IL-6/STAT3 [46, 84, 85].

### Notch

The Notch pathway is highly conserved in various organisms, is involved in developing almost all organ systems, and regulates tissue homeostasis after development. The Notch signaling pathways include Notch ligands (Delta-like ligands 1, 3, 4, Serrate-like ligands Jagged1, Jagged2), Notch1, Notch2, Notch3, Notch4, Notch DNA binding proteins, immunoglobulin κJ region recombinant signaling proteins and effector molecules (Hes, Hcy, Herp) [86]. The notch signaling pathway activates Notch receptors on the cell surface upon binding to ligands, inducing protein hydrolase cleavage. The intracellular segment of Notch is released into the nucleus and binds to the transcriptional repressor RBP-Jκ to activate the transcription of target genes, which can regulate cell proliferation, cell differentiation, and apoptosis [87]. In the DSS-induced UC mice, Gegen Qinlian Decoction maintains mucosal homeostasis through bidirectional regulation of Notch signaling, thereby restoring colonic epithelial function [88]. Compound Sophorae Decoction modulates the Notch signaling, promotes altered macrophage phenotype, and enhances colonic mucosal barrier function [89]. QingBai Decoction inhibited the effects of NF-κB and Notch signaling on the inflammatory cascade, which effectively alleviated intestinal inflammation and mucosal barrier function in DSS-induced UC mice [13].

### Others

TLRs are vital pattern recognition receptors that can lead to uncontrolled inflammatory responses in case of excessive activation [90]. IL-1 receptor-associated kinase (IRAK) mediates multicellular receptor signaling, including TLRs, and has an essential regulatory role in various inflammatory cell signaling networks [91]. Tiaochang Xiaoyan Extract Tablets improved colonic inflammation in rats with chronic colitis, and this effect may be achieved by activating lysosomes in macrophages through inhibition of the TLR9/MyD88/IRAK signaling pathway [92].

The primary biological function of Oncostatin-M (OSM) is to inhibit the growth of a variety of tumor cells and to induce the differentiation of specific tumor cells. OSM is a promising cytokine because it can significantly inhibit the growth and induce the differentiation of tumor cells through a variety of different pathways.

The Oncostatin-M receptor (OSMR) is widely distributed on the surface of many tumor cells, endothelial cells, and epithelial cells [93]. The expression of OSM and OSMR is increased in intestinal tissues of patients with IBD, which leads to increased intestinal inflammation. Therefore, OSM and OSMR have become markers in IBD diagnosis [94]. Feiyangchangweiyan Capsule affects UC by inhibiting the OSM/OSMR pathway and regulating inflammatory factors to improve intestinal flora [95].

The Wnt signaling pathway regulates the growth and development of the body, which maintains the normal physiological functions of many tissues and organs, and its abnormal activation or inhibition contributes to the development of many diseases. The Wnt/β-catenin signaling pathway is one of the most important signaling pathways that significantly impact the progression of multiple diseases. Its central role is to activate the proliferation of intestinal stem cells and inhibit the differentiation of intestinal stem cells from maturing cell types [96, 97]. Sishen Pill effectively attenuated TNBS-induced colitis, which inhibited the Wnt/β-catenin signaling pathway [77].

## Oxidative stress

It has been reported that the concentration of reactive oxygen species (ROS) in intestinal cells increases during chronic inflammatory and recurrent immune responses in the gut as the disease develops [98]. ROS regulates cellular regulation by oxidizing DNA, proteins, lipids, and other cellular structures. In order to protect biological systems from damage caused by excessive ROS, the cellular antioxidant system is activated and thus regulates ROS production. ROS levels were significantly increased in the colon of DSS-induced UC mice. Gegen Qinlian decoction and Sanhuang Shu'ai decoction reduced ROS concentrations and thus prevented the onset and progression of the disease [60, 99]. Nuclear factor E2-related factor 2 (Nrf2) is an important transcription factor regulating the cellular oxidative stress response and is a central regulator in maintaining intracellular redox homeostasis. Nrf2 regulates constitutive and inducible expression of a series of antioxidant proteins to mitigate reactive oxygen species and electrophilic body-induced cellular damage and maintain tissue cell redox dynamic homeostasis [100]. Huang-Lian-Jie-Du Decoction can effectively alleviate DSS-induced UC mice by inhibiting the NF-κB signaling pathway, activating the Nrf2 signaling pathway, and enhancing intestinal barrier function [14]. Superoxide dismutase (SOD), catalase (CAT), and glutathione (GSH) are downstream enzymes of the Nrf2 pathway that convert oxygen radicals into hydrogen peroxide and rapidly break them down into the water, effectively preventing tissue cells from being damaged by peroxides [101]. SOD can scavenge superoxide anions produced in the body. CAT is a crucial component of the enzyme system in the body’s antioxidant system, which can scavenge free radicals in synergy with SOD, and GSH can protect cell membrane structure and function from oxidative damage by hydrogen peroxide. All SOD, CAT, and GSH have essential roles as antioxidants, and when their levels and activity are reduced, they lead to the accumulation of free radicals [102]. Myeloperoxidase (MPO) is a crucial peroxidase produced by neutrophil asplenophil granules, and its activity level reflects the degree of neutrophil infiltration. Malondialdehyde (MDA) is the end product of the lipid peroxidation reaction between ROS and unsaturated fatty acids on the cell membrane, and its level can indirectly reflect the degree of lipid peroxidation [102]. Gegen Qinlian Decoction and Kuijieyuan Decoction reduced MDA and MPO levels and increased SOD, GSH, and CAT levels, showing a protective function against UC animal models [60, 61].

## Novel Chinese herbal medicine formula

As a unique health resource in China, Chinese Medicine is increasing attention worldwide. In novel drug development, Chinese Herbal Medicine and natural products have become an essential source of innovative drug development. At present, for the research and evaluation of new drugs in different categories of Chinese Medicine and natural drugs, based on many years of experience in new drug research in Chinese medicine, many people have proposed ideas for the research and evaluation of drugs in different categories originating from classical formulas, and combined with basic research to carry out innovative research in various aspects.

Qingre Jianpi Decoction, the classically formulated optimized Qingchang Suppository, and the simplified prescription Suqing Pill reduced the secretion of inflammatory cytokines and exerted anti-inflammatory effects in UC treatment [103–105]. The Chinese herbal standardized product Guchang Capsule, Semi-bionic Extraction of compound Turmeric, Modified Pulsatilla Decoction, the functional beverage Ampelopsis grossedentata, and Guchang Zhixie Pill can all exert protective effects against IBD via the NF-κB signaling pathway [106–110].

Chemokine receptor 3 (CXCR3) is a specific binding protein that inhibits the endogenous chemokine IP10, and the IP10/CXCR3 axis plays a vital role in the pathogenesis of childhood IBD, and inhibition of IP10 may alleviate the clinical symptoms of UC [111]. CRCX3 is activated once it binds to IP10 and contributes to its transfer to the inflammatory localization for further effects. The large amount of inflammatory cytokines produced by local inflammation of intestinal mucosa stimulates IP10 to recruit more inflammatory cells to form a cascade response and aggravates the damage of the intestinal mucosal barrier [112]. Qingchang Wenzhong Decoction was able to downregulate IP10/CXCR3 axis-mediated inflammatory response, improve DSS-induced UC in rats, and maybe a new UC therapy [113]. Aquaporins (AQPs) are essential proteins in the body's aqueous metabolism process, mediating the transport of water molecules along an osmotic gradient across cell membranes and mediating the transport of water molecules across different cell membranes [114]. AQPs are mainly involved in regulating urine concentration and fluid permeation in humans [115]. Ershen Pill Extract effectively improved diarrhea in rats by improving the synthesis of AQP3 in the colon [116]. In DSS-induced colitis, the increase in vascular permeability precedes the increase in intestinal epithelial permeability. Qingchang Suppository can reduce colonic vascular permeability and improve vascular endothelial barrier function by modulating the VEGF/HIF-1α signaling pathway [117]. In addition, some novel herbal combinations play an essential role in IBD. The use of Qingchang Suppository reduced colonic tissue edema, vascular congestion, and inflammatory cell infiltration [117]. Costus root granules significantly improved inflammation and apoptosis in the colonic epithelium by modulating the transforming growth factor (TGF)-β-mediated PI3K/AKT signaling pathway [118]. Xinhuang Tablets alleviated DSS-induced UC in mice by increasing intestinal epithelial TJ expression [119]. Composite Sophora Colon-Soluble Capsule also showed significant effects on UC from restoring intestinal microbiota and intestinal immune homeostasis [120].

The CHMF prepared by different methods can provide vital information for further mechanistic exploration of traditional prescriptions, contributing to the rational application of herbal compounding in modern applications or scientific research and improving human knowledge of herbal compounding. Banxia Xiexin Decoction is widely used in modern clinical practice. Both modern and ancient extraction methods can alleviate the severity of UC rats to different degrees [121]. The differences in the efficacy of the five Ganjiang Decoction extracts on DSS-induced UC in mice were closely related to the extraction methods. The study by Wei improved the extraction process of Ganjiang Decoction, which provided the basis for the process of enteric preparation and offered new ideas for the compounding of Chinese Herbal Medicines [122]. These Novel Chinese Herbal Medicine Formulas provide reference and reference for the development of new drugs in Chinese Medicine and natural products, intending to improve the efficiency and success rate of new drug development and enhance the development of new products.

## Conclusions

The development of new drugs for CHMF is a crucial area of modern research in Chinese medicine. Chinese medicines, especially CHMF, are different from chemical, biological, and botanical drugs. Due to the complexity of the pharmacological substances and targets or links contained in CHMF, it poses a great difficulty for new drug development, but at the same time, it also has significant room for innovation. At present, the clinical treatment of IBD is mainly based on immunosuppressive drugs or hormone therapy, and the use of these drugs is strictly regulated, which is prone to adverse reactions and drug resistance if used irregularly. Table 1 shows the targets of the Chinese Herbal Medicine Formula in attenuating IBD. We hope to explore the association between CHMF and IBD to find new ideas for new drug development. At present, we should be oriented to highlight the characteristics and advantages of CHMF efficacy, fully utilize and draw on modern biotechnology, and thoroughly study the scientific and technological issues related to CHMF development. The level of knowledge of CHMF pharmacological substances and mechanisms of action should be further understood to contribute to creating high-level new drugs and provide new directions and ideas for the treatment of IBD.

**Table 1:** The targets of the Chinese Herbal Medicine Formula in attenuating IBD

Chinese herbal medicine formula	Components	Experiment models	Targets	References
*Indirubin* and *Isatin*	*Isatis indigotica* Fort., *Baphicacanthus cusia* (Nees) Bremek. and *Polygonum tinctorium* Ait	DSS-induced UC mice	Reduce pro-inflammatory mediators and MPO, increase anti-inflammatory cytokines and Foxp3, inhibit CD4+ T cell infiltration, inhibit oxidative stress and epithelial cell apoptosis, inhibit NF-κB and MAPK pathways	[7]
Fuzi-Ganjiang	*Aconitum carmichaelii* Debeaux and *Zingiber officinale* Roscoe	DSS-induced UC mice	Inhibit MPO and inflammatory cytokines, inhibit the activation of MAPK, NF-κB and STAT3 signaling pathways	[8]
*Astragalus membranaceus* polysaccharides and *Codonopsis pilosula*	Total polysaccharides of *A. membranaceus* extractum and the *C. pilosula* extractum	DSS-induced UC mice	Rebuild immune balance, alleviate colonic mucosal damage, activate AhR, up-regulate isovaleric acid and butyric acid, and restore intestinal flora structure	[9]
QingBai decoction	Indigowoad Leaf, Indigowoad Root, Amur Corktree Bark, Lightyellow Sophora Root, Coix Seed, Cuttlebone	DSS‐induced UC mice	Up-regulate the expression of tight TJs and mucus 2, reduce the production and secretion of pro-inflammatory cytokines, and modulate NF-κB and Notch pathways	[13]
Huang-Lian-Jie-Du Decoction	*Coptidis Rhizoma*, *Scutellariae Radix*, *Phellodendri Chinensis Cortex* and *Gardeniae Fructus*	DSS-induced UC mice	Inhibit NF-κB signaling pathway, activate Nrf2 signaling pathway, enhance intestinal barrier, inhibit NF-κB signaling pathway, activate Nrf2 signaling pathway, enhance intestinal barrier function, inhibit the growth of intestinal pathogens, prevent the reduction of beneficial bacteria, correct the dysfunction of intestinal flora	[14, 24, 34, 83]
Huangqin Decoction	*Scutellaria baicalensis* Georgi, *Paeonia lactiflora* Pall, *Glycyrrhiza uralensis* Fisch, and *Ziziphus jujuba* Mill	DSS-induced UC mice TNBS-induced UC mice	Inhibit inflammation, alter gut microbiota, modulate SCFAs, prevent cellular damage caused by oxidative stress, inhibit Ras-PI3K-Akt-HIF-1α and NF-κB pathways	[15, 23, 35, 36, 79]
Qing Hua Chang Yin	*Coptis chinensis* Franch, Herba et Gemma Agrimoniae, Radix Sanguisorbae, *Magnolia officinalis*, Radix Paeoniae Rubra, *Elettaria cardamomum*, Semen Coicis, *Artemisia capillaris* Thunb, Semen Dolichoris Album, Herba Eupatorii Fortunei and *Poria cocos*	LPS-induced Caco-2 cell	Regulate TJs protein expression, protect intestinal epithelial barrier integrity after inflammatory injury	[16]
Sijunzi Decoction	*Ginseng Radix et Rhizoma*, or *Codonopsispilosula*, *Atractylodes Macrocephalae Rhizoma*, *Poria*, and *Glycyrrhizae Radix et Rhizoma Praeparatecum Melle*	TNBS-induced UC mice	Claudin-2, NF-κB and MLCK signaling pathway	[17]
*Pyungwi-san*	*Citri Pericarpiumpeel*, *glycyrrhizin*, *magnolol*, *Rhizoma Atractylodis Macrocephalae*	DSS-induced UC mice	Modulate TJ protein, ameliorate pro- and anti-inflammatory cytokines, modulate gut microbiota, regulate TLR4 and PPARγ expression, inhibit NF-κB pathway and inhibit the activation of NLRP3 inflammasome	[25, 27, 73]
Huai Hua San	Flos Sophorae (the dried flower bud of *Sophora japonica* L.), Cacumen Platycladi (the dried branches and leaves of *Platycladus orientalis* (L.) Franco), Fructus Aurantii (the dried immature fruit of *Citrus aurantium* L.) and Herba Schizonepetae (the dried overground parts of *Schizonepeta tenuifolia* Briq.)	DSS-induced IBD rats	Improve dysbiosis of the microbiome at the taxonomic level	[26]
Compound Sophorae Decoction	*Radix sophorae flavescens*, *Radix sanguisorbae*, *Rhizoma bletillae*, *Radix glycyrrhizae*, and *Indigo naturalis*	TNBS-induced UC rats DSS-induced IBD mice	Regulate the levels of metabolic biomarkers, reduce epithelial cell apoptosis, promote epithelial cell regeneration, up-regulate TJs expression and MUC2 secretion, modulate Notch signaling, reduce M1/M2 ratio, reduce inflammatory factors, and regulate Th17/Treg balance	[32, 47, 89]
Huankuile Suspension	Trukish galls, Coptis chinensis, pomegranate flower, amber, tabasheer and plantain herb	TNBS-induced UC rats	Reduce pro-inflammatory cytokines, modulate bile metabolism, pyrimidine metabolism, purine metabolism, glutathione metabolism and citric acid cycle	[33]
Rhubarb Peony Decoction	*Rhei Radix et Rhizoma*, *Moutan cortex*, *Persicae semen*, *Natrii sulfas*, and *Benincasae semen*	DSS-induced UC mice	Altered gut microbiota, restored SCFA content in the gut, and modulate the ratio of Th17 cells to Treg cells	[37]
Sishen Pill	*Semen psoraleae*, *Fructus evodiae*, *Semen myristicae* and *Schisandra chinensis*	DSS-induced UC mice TNBS-induced UC rats	Effectively control Tem cells in peripheral blood, inhibit PI3K/Akt signaling pathway, modulate the interaction between inflammatory DCs and gut microbiota, inhibit Wnt/β-catenin signaling pathway, inhibit the activation of NF-κB by NEMO/NLK signaling pathway	[39, 49, 69, 77]
Gegen Qinlian Decoction	Berberine, Baicalin, and Puerarin	DSS-induced UC mice	Inhibit IL-6/JAK2/STAT3 signaling, restore Treg and Th17 cell balance, reduce phagocytic cell differentiation, promotes CBC proliferation, reduce Notch-activated Hes1 protein in HT29 and FHC cells, and increase Notch inhibition Hes1 protein in cells, inhibit TLR4/NF-κB signaling and enhance antioxidant effects	[45, 60, 88]
Qingre Zaoshi Liangxue Decoction	*Sophora flavescens* Aiton., *Bletilla striata* Rchb. f., *Glycyrrhiza uralensis* Fish., *Coptis chinensis* France., *Baphicacanthus cusia* (Nees) Bremek	TNBS-induced UC mice	Decrease FICZ concentration and AhR signaling in the colon, decrease expression of IL-6, STAT3 and RORγt, increase expression of FOXP3, decrease the proportion of Th17 cells and increase the proportion of Treg cells	[46]
Bawei Xileisan	Watermelon frost, calcite, cow gallstone, pearl powder, borax, borneol (*Dryobalanops aromatica* Gaertn. f.), ammonium chloride, and *Indigo naturalis*	DSS-induced UC mice	Restore Th17/Treg balance, improve fecal Lactobacillus levels, and protect gut microbiota	[48]
Huangkui Lianchang Decoction	*Abelmoschus manihot* (L.) *Medik*, *Euphorbia humifusa Willd*, *Pteris multifida Poir*, *Lithospermum erythrorhizon Siebold* & *Zucc*, *Rubia cordifolia* L., and *Rhus chinensis* Mill	DSS-induced UC mice	Alleviate colon pathological damage, reduce MPO and SOD activities, and inhibit NF-κB signaling pathway	[53]
Tou Nong San	*Radix astragali*, *Angelica sinensis*, *Ligusticum*, Spina Gleditsiae, and pangolin scales	TNBS-induced UC rats	Inhibit NF-κB signaling pathway and regulate pro-inflammatory cytokines	[55]
Jian-Pi Qing-Chang Decoction	*Astragalus*, *Codonopsis pilosula*, *Portulaca oleracea*, *Sanguisorba officinalis*, *Notoginseng*, *Bletilla striata*, *Radix Aucklandiae*, and *Licorice*	DSS-induced UC mice	Improve mucosal inflammatory response and intestinal epithelial barrier function through NF-κB/HIF-1α signaling pathway, reduce the expression of IL-1β, IL-8 and TNF-α, activation of NF-κB and phosphorylation of IκB were significantly inhibited	[56, 57]
Kuijieyuan Decoction	*Astragalus mongholicus* Bunge, *Hedyotis diffusa* Willd., *Cirsium undulatumn* (Nutt.) Spreng, *Cirsium setosum* (Willd.) M. Bieb., *Pulsatilla vulgaris* Mill, *Prunella vulgaris* L. subsp. Vulgaris, *Coptis chinensis* Franch, *Polygonum cuspidatum* Sieb. et Zucc., *Atractylodes lancea* (Thunb) DC and *Glycyrrhiza glabra* L.	DSS-induced UC rats	Inhibit TLR4/NF-κB signaling pathway, inhibit PI3K/AKT/NF-κB signaling pathway and oxidative stress	[61]
Bu-Zhong-Yi-Qi Granule	Astragalus, *Codonopsis pilosula* (Franch.) Nannf., Liquorice, *Atractylodes macrocephala*, Tangerine peel, Cohosh, Bupleurum, Angelica, Zingiber officinale Roscoe, and Jujube	TNBS-induced UC rats	Restore the expression of TJ protein and regulate the secretion of some inflammatory cytokines through the TLR4/NF-κB/MLCK pathway	[64]
Shen-Ling-Bai-Zhu-San	*Panax ginseng C. A. Mey.*, *Poria cocos* (Schw.) Wolf, *Atractylodes macrocephala* Koidz., *Dioscorea opposita* Thunb., *Dolichos lablab* L., *Nelumbo nucifera* Gaertn., *Coix lacryma-jobi* L. var. mayuen. (Roman.) Stapf, *Amomum villosum* Lour., *Platycodon grandiflorum (Jacq.) A. DC*, and *Glycyrrhiza glabra* L.	DSS-induced UC mice	Inhibit MAPK/NF-κB signaling pathway	[65]
Jiaweishaoyao Decoction	*Radix Et Rhizoma Glycyrrhizae*, *Semen Arecae*, *Magnolia officinalis Rehd et Wils*, *Crataegus pinnatifida Bunge*, *Radix Et Rhizoma Rhei*, *Massa Medicata Fermentata*, *Radix Paeoniae Alba*, and *Radix Angelicae Sinensis*	DSS-induced UC mice	Inhibit NLRP3 inflammasome and NF-κB pathway	[72]
Zuojin Pill	*Coptis chinensis* and *Evodia rutaecarpa*	DSS-induced UC mice	Regulate gut microbiota, improve CD4^+^CD25^+^Foxp3^+^ and PD-L1^+^ Treg cells, inhibit PI3K/Akt signaling pathway	[76]
Xianglian Pill	*Coptidis Rhizoma* and *Aucklandiae Radix*	DSS-induced UC mice	Inhibit the secretion of pro-inflammatory cytokines, repair the dysfunction of the intestinal epithelial barrier, enhance autophagy, block the activation of the PI3K/Akt/mTOR pathway, weaken the protective effect of XLP on colitis, block the activation of PI3K/Akt/mTOR signaling pathway to promote autophagy	[78]
Pien Tze Huang	*Moschus*, *Calculus bovis*, *Snake gall* and *Radix notoginseng*	DSS-induced UC mice	Suppress levels of inflammatory biomarkers and inhibit IL-6/STAT3 signaling, improve gut microbiota	[84]
Baitouweng Decoction	*Radix pulsatilla*, *Cortex phellodendri*, *Rhizoma coptidis*, and *Cortex fraxini*	DSS-induced UC mice	Regulate gut microbiota and IL-6/STAT3 inflammatory signaling pathway	[85]
Tiaochang Xiaoyan Extract Tablets	*Radix Astragali*, *Lindera aggregata*, *Rhizoma coptidis*, *Oldenlandia diffusa* and *coix seed*	TNBS-induced UC rats	Repair of colonic mucosal damage, reduce inflammation, increase lysosomal activity of macrophages, and decreased DAI in rats with colitis, improved colonic inflammation and infiltrate CD11c^+^ macrophages in rats with chronic colitis, and inhibit TLR9/MyD88/IRAK signaling pathway	[92]
Feiyangchangweiyan Capsule	*Euphorbia hirta* L., *Polygonum chinense* L., and *Ilex rotunda* Thunb	DSS-induced UC mice	Inhibit OSM/OSMR pathway and modulate inflammatory factors, modulate gut microbiome composition	[95]
Sanhuang Shu'ai decoction	*Coptidis Rhizoma*, *Scutellariae Radix*, *Phellodendri Chinensis Cortex*, and *Artemisiae Argyi Folium*	DSS-induced UC mice	Reduce oxidative stress, modulate gut microbiota, reduce inflammatory mediators and cytokines	[99]
Qingre Jianpi Decoction	*Radix Astragali Mongolici*, *Rhizoma Coptidis*, *Herba Violae Philippicae*, *Radix Paeoniae Alba*, *Semen Alpiniae Katsumadai*, *Radix Notoginseng*, *Semen Plantaginis* and *Semen Myristicae*	DSS-induced UC mice	Reduce the secretion of inflammatory cytokines, inhibit the activation of the NLRP3 inflammasome, and inhibit the inflammatory infiltration of immune cells	[103]
Qingchang Suppository	*Radix Notoginseng*, *Indigo Naturalis*, *Gallnut*, Herba Portulacae and borneol	TNBS-induced UC rats DSS-induced UC rats	Improve colonic hypoxia, reduce the expression of VEGF, HIF-1α and iNOS, reduce colonic VP, modulate VEGF/HIF-1α signaling pathway to improve vascular endothelial barrier function, and inhibit JAK2/STAT3 pathway	[104, 117]
simplified prescription Suqing Pill	*Lonicera japonica* Thunb., *Forsythia suspensa* (Thunb.) Vahl, *Taraxacum mongolicum* Hand. -Mazz., *Sanguisorba officinalis* L., *Trichosanthes kirilowii* Maxim., *Angelica dahurica* (Fisch. ex Hoffm.) Benth. et Hook. f., *Rehmannia glutinosa* Libosch., *Cimicifuga heracleifolia* Kom., *Astragalus membranaceus* (Fisch.) Bge. var. *mongholicus* (Bge.) Hsiao, *Angelica sinensis* (Oliv.) Diels, *Callus gallus domesticus* Brisson, *Scrophularia ningpoensis* Hemsl, and *Glycyrrhiza uralensis* Fisch	DSS-induced UC mice	Reduce the level of inflammatory factors, increase the level of anti-inflammatory cytokines, down-regulate oxidative factors	[105]
Guchang Capsule	Halloysite, *Coptis*, *Phellodendron amurense*, Myrobalan, Nutmeg, *Cortex Magnoliae officinalis*, Fructus Evodiae, Qu Jian, Cinnamon, Rhizoma Zingiberis, Chinese Prickly Ash, *Ligusticum wallichii*, Concha Ostreae, Gallnut, and Dark Plum Fruit	DSS-induced UC mice	Inhibit NF-κB activation in macrophages and reduce pro-inflammatory cytokine production	[106]
Semi-bionic Extraction of compound Turmeric	*Curcuma wenyujin Y, H. Chen et C. Ling, Terminalia chebula Retz., Scutellaria baicalensis Georgi*, *Rheum palmatum* L., *Coptis chinensis* Franch., *Phellodendron amurense* Rupr., *Gardenia jasminoides J. Ellis*, and *Paeonia lactiflora Pall*	DSS-induced UC rats	Reduce inflammatory factors and increased anti-inflammatory factors, alleviate histological changes in colon, inhibit the activation of NF-κB and ICAM-1	[107]
Modified Pulsatilla Decoction	*Radix Pulsatillae*, *Cortex Phellodendri*, *Rhizoma Coptidis*, *Cortex Fraxini*, *Sanchi*, *Radix Paeoniae Rubra*, and *Radix Glycyrrhizae*	Oxazolone-induced UC mice	Inhibit the activation of NF-κB signaling pathway and reduce the severity of colitis	[108]
Ampelopsis grossedentata	A large amount of flavonoid active ingredients	DSS-induced UC mice	Inhibit IRAK1/TRAF6/NF-κB-mediated inflammatory signaling pathway, inhibit the elevated expression levels of TNF-α, IL-1β, IL-6 and IL-8	[109]
Guchang Zhixie Pill	*Coptidis Rhizoma*, *Zingiberis Rhizoma*, *Corydalis Rhizoma*, *Mume Fructus*, *Aucklandiae Radix* and *Papaveris Pericarpium*	DSS-induced UC rats	Inhibit the STAT3/NF-κB/IL-6 pathway, enrich in inflammation, immunity and oxidative stress-related pathways	[110]
Qingchang Wenzhong Decoction	*Coptis*, Zingiber officinale Roscoe, *matrine*, *Indigo*, *sanguisorba carbon*, wood, *pseudoginseng* and *licorice*	DSS-induced UC rats	Decrease DAI, HS and MPO levels, down-regulate of IP10/CXCR3 axis-mediated inflammatory response	[113]
Ershen Pill Extract	*Myristica Fragrans* and *Fructu Psoraleae*	Disorder-Diet-induced Pi yang deficiency diarrhea	Improve AQP3 synthesis in the colon	[116]
Costus root granules	The extract of Costus root, ingredient dissolution into a traditional water decoction	TNBS-induced UC rats	Inhibit the apoptosis of colonic epithelial cells, increase the expression of TGF-β, and activate the PI3K/AKT signaling pathway	[118]
Xinhuang Tablets	*Herba Sarcandrae*, *Radix Notoginseng*, *Calculus Bovis* and so on	DSS-induced UC mice	Reduce circulating levels of TNF-α and SAA, increase protein levels of TJs, and decrease phosphorylation levels of Elk-1	[119]
Composite Sophora Colon-Soluble Capsule	Radix Sophorae Flavescentis, Indigo Naturalis, *Bletilla striata*, Radix Sanguisorbae, and Licorice Root	DSS-induced UC mice	Restore gut microbiota, restore gut immune balance, reduce Th17 cell numbers, and increase the percentage of Treg cells	[120]
Banxia Xiexin Decoction	*Rhizoma*, *Coptidis Rhizoma*, *Scutellariae Radix*, *Zingiberis Rhizoma*, *Ginseng Radix*, *Jujubae Fructus*, and *Glycyrrhizae Radix*	TNBS-induced UC rats	Anti-inflammatory	[121]
Ganjiang Decoction	*Zingiberis Rhizoma*, A*ngelicae Sinensis Radix*, *Coptidis Rhizoma*, P*hellodendri Chinensis Cortex*, *Sanguisorbae Radix*, *Granati Pericarpium*, and *Asini Corii Colla*	DSS-induced UC mice	Improve DAI score, colon length, relative spleen weight, pathological analysis results, and inflammatory factors	[122]

In the West, biological agents are the fastest-growing segment of the prescription drug market and cost thousands of dollars per patient per year to treat IBD. There is a need to reconcile the most appropriate treatment for these patient populations from the perspectives of both disease presentation and cost. In developing countries, healthcare systems and indeed the patients struggle to afford such expensive treatments [123].

The classics of traditional Chinese medicine are summaries of the rich practical experience of physicians in the past dynasties, and they are the standard to guide the clinical practice of traditional Chinese medicine for thousands of years. By collecting and arranging medical records and herbal medicines of past dynasties, combined with folk prescriptions and prescriptions offered by famous doctors, traditional Chinese medicines and their compound prescriptions with apparent curative effect, wide clinical application, apparent characteristics, and advantages are sorted out. On this basis, the experimental research of screening traditional Chinese medicines with various pharmacological models is carried out, and modern multidisciplinary technology is used to reveal the pharmacodynamic material basis and molecular mechanism of traditional Chinese medicines and then promote the development of innovative traditional Chinese medicines and their application in clinical practice. For example, arsenic trioxide, developed from the traditional Chinese medicine arsenic for treating acute promyelocytic leukemia (APL), has a good effect [124]. The excavation and development of classic prescriptions is the entry point and breakthrough point for the inheritance and innovation of traditional Chinese medicine in the new era. The classic recipes derived from ancient books have thousands of years of human experience and are widely used in common diseases, frequently-occurring diseases, chronic diseases, and other fields. The research and development of classic prescriptions is a fast and effective transformation path, effectively filling the gaps in medical drugs for some chronic and intractable diseases. The commonly used CHMF originates from clinical practice and has experienced thousands of years of inheritance and practice. However, these CHMFs are challenging to be recognized by the public at home and abroad. For the widely used clinical prescriptions with exact curative effects, the world-recognized methods and scientific data are used to demonstrate their clinical efficacy and safety. Their clinical positioning is precise, which is conducive to the broader and more effective clinical use of traditional Chinese medicine.

The elucidation of the material basis of compound recipes is of great significance to the research on the mechanism of action of traditional Chinese medicine, quality control, new drug research, and new drug development. In recent years, modern scholars, based on the holistic view of traditional Chinese medicine, combined the spectrum-effect relationship of traditional Chinese medicine theory and bioinformatics to clarify the pharmacodynamic substances as well as their mechanism of action and achieved a transformation. Identify the pharmacodynamic substances, explain the interaction between CHMF and the body, and then use pharmacological methods to study the mechanism of action. Considering a single factor's physical and chemical factors, the traditional Chinese medicine etiological animal model induced by biological damage, the Western medicine etiological and pathological animal model, and the clinical application characteristics of Chinese medicine cannot be well integrated. The researchers further established a disease-syndrome combination model to study the material basis and mechanism of CMHF. The combination of disease and syndrome model can reflect the characteristics of traditional Chinese medicine and be supported by the pathological changes and diagnostic evaluation of diseases in Western medicine. It is a trend in modern Chinese medicine research models. In this way, the pharmacodynamic material basis and mechanism of CHMF can be analyzed from the clinical point of view, the nature of the disease, and the overall composite level.

## Data Availability

Not applicable.

## Abbreviations

IBD: Inflammatory bowel disease
CD: Crohn’s disease
UC: Ulcerative colitis
CHMF: Chinese herbal medicine formula
IECs: Intestinal epithelial cells
TJ: Tight junction
ZO: Zonula occludens proteins
DSS: Dextran sulfate sodium
LPS: Lipopolysaccharide
TNBS: 2,4,6-Trinitrobenzene sulfonic acid
CDD: Clostridium difficile
SCFAs: Short-chain fatty acids
GALT: Gut-associated lymphoid tissue
DCs: Dendritic cells
Tfh: T follicular helper
IL: Interleukin
TGF: Transforming growth factor
NF-κB: Nuclear factor kappa B
IκB: Inhibitor of the NF-κB
IKK: Inhibitor of NF-κB kinase
TLRs: TOLL-like receptors
MyD88: Myeloid differentiation factor 88
MLCK: Myosin light chain kinase
MLC: Myosin light chain
MAPK: Mitogen-activated protein kinase
ERK: Extracellular regulated protein kinase
JNK: Jun N-terminal kinase
IκBKG: κ Light chain polypeptide repressor gene
NLK: Nemo-like kinase
nlrP3: NOD-like receptor protein 3
ASC: Apoptosis-associated Speck-like protein containing a CARD
JAK: Janus kinase
STAT3: Signal transducer and activator of transcription 3
TNF-α: Tumor necrosis factor-α
IRAK: IL-1 receptor-associated kinase
OSM: Oncostatin-M
OSMR: Oncostatin-M receptor
ROS: Reactive oxygen species
Nrf2: Nuclear factor E2-related factor 2
SOD: Superoxide dismutase
CAT: Catalase
GSH: Glutathione
MPO: Myeloperoxidase
MDA: Malondialdehyde
AQPs: Aquaporins
